# What Are the Effects of Vitamin A Oral Supplementation in the Prevention and Management of Viral Infections? A Systematic Review of Randomized Clinical Trials

**DOI:** 10.3390/nu14194081

**Published:** 2022-10-01

**Authors:** Alessandra Sinopoli, Susanna Caminada, Claudia Isonne, Maria Mercedes Santoro, Valentina Baccolini

**Affiliations:** 1 Department of Prevention, Local Health Authority Roma 1, 00193 Rome, Italy; 2 Department of Experimental Medicine, University of Rome “Tor Vergata”, 00133 Rome, Italy; 3 Department of Public Health and Infectious Diseases, Sapienza University of Rome, 00185 Rome, Italy

**Keywords:** Vitamin A, viral infections, micronutrients, systematic review

## Abstract

Vitamin A (VA) deficiency is associated with increased host susceptibility to infections, but evidence on its role in the prevention and management of viral infections is still lacking. This review aimed at summarizing the effects of VA supplementation against viral infections to support clinicians in evaluating supplemental treatments. PubMed, Scopus, and Web of Science were searched. Randomized clinical trials comparing the direct effects of VA oral supplementation in any form vs. placebo or standard of care in the prevention and/or management of confirmed viral infections in people of any age were included. A narrative synthesis of the results was performed. The revised Cochrane Risk-Of-Bias tool was used to assess quality. Overall, 40 articles of heterogeneous quality were included. We found data on infections sustained by *Retroviridae* (*n* = 17), *Caliciviradae* (*n* = 2), *Flaviviridae* (*n* = 1), *Papillomaviridae* (*n* = 3), *Pneumoviridae* (*n* = 4), and *Paramyxoviridae* (*n* = 13). Studies were published between 1987 and 2017 and mostly conducted in Africa. The findings were heterogeneous across and within viral families regarding virological, immunological, and biological response, and no meaningful results were found in the prevention of viral infections. For a few diseases, VA-supplemented individuals had a better prognosis and improved outcomes, including clearance of HPV lesions or reduction in some measles-related complications. The effects of VA oral supplementation seem encouraging in relation to the management of a few viral infections. Difference in populations considered, variety in recruitment and treatment protocols might explain the heterogeneity of the results. Further investigations are needed to better identify the benefits of VA administration.

## 1. Introduction

The term “Vitamin A” (VA) refers to a group of fat-soluble retinoids, including retinol, retinal and retinyl esters [1]. Animals are incapable of de novo VA synthesis; therefore, dietary VA is obtained in the diet as preformed VA from animal sources, or as provitamin carotenoids such as beta-carotene from plant sources. Specifically, preformed VA can be obtained mostly from dietary animal sources as retinyl palmitate, whereas among carotenoids obtained only from plant sources [2], β-carotene is the most represented. Retinol is absorbed from the digestive tract while carotene is taken up by enterocytes by the membrane transporter protein scavenger receptor B1. Retinol is esterified to retinyl esters and stored in the stellate cells in the liver. Retinol and beta-carotene are therefore oxidized to retinal and retinoic acid in the tissues [1]. Good dietary sources of provitamin carotenoids include carrots and other dark-colored fruits such as mangoes, oranges, cantaloupe [3]. VA plays an essential role in many physiological functions, including vision, growth, reproduction, hematopoiesis immunity and cellular integrity [4].

VA deficiency is associated with increased host susceptibility to infections [5]. Green in 1928 was the first to introduce VA as “anti-infective vitamin” [6]. Later studies have clarified that VA promotes recovery from infection rather than prevention of infection [7,8,9]. In particular, in vitro techniques have demonstrated that VA plays a crucial role in the establishment and maintenance of the human immune system [10,11]. Additionally, human research shows that there is a correlation between a deficiency of micronutrients (particularly VA) and infectious diseases spread through the respiratory and digestive systems in children [12].

Although in the 1940s the advent of antibiotics [13] dampened the interest in the research of substances with antiviral properties, the recent COVID-19 pandemic rekindled the attention on this research field. At the present time, there is no convincing evidence that demonstrates a role for vitamin supplementation or other natural supplements in the fight against COVID-19: some positive results against viral infections have been provided for Vitamin B, especially B9 and B12 [14], Vitamin C [15], Vitamin D [16] and other substances such as lactoferrin [17], but evidence on the potential effects in the prevention and management of viral infections from clinical studies is still fragmented for VA. The aim of our systematic review was to identify the direct effects of orally administered VA against viral infections in adults and children to provide a synthesis of the results and support clinicians in the evaluation of supplemental treatments for viral diseases.

## 2. Materials and Methods

This systematic review was conducted according to the Cochrane Handbook for systematic reviews and the Preferred Reporting Items for Systematic Reviews and Meta-Analyses (PRISMA) statement [18]. The review protocol was registered at PROSPERO, identifier CRD42022338234. Because this study did not involve primary data collection, the protocol was not submitted for institutional review board approval and did not require informed consent.

### 2.1. Inclusion and Exclusion Criteria

Eligible articles were randomized clinical trials (RCTs) conducted in any country, published in English or Italian, that compared data on the direct effects of orally administered VA in any form including preformed VA, such as retinol or retinyl esters (e.g., retinyl acetate or retinyl palmitate), and provitamin A carotenoids, such as beta-carotene, vs. placebo or standard of care in the prevention and/or management of confirmed viral infections in people of any age. When VA was given in combination with other substances (e.g., multivitamins), the study was considered eligible only when the VA effect could be isolated (e.g., multivitamins including VA vs. multivitamins excluding VA). No minimum VA dosage was required. Any virus family was considered eligible.

Any indirect effect, such as the outcomes of VA supplementation in children born from women receiving the nutrient, was excluded. We also excluded articles using in vitro techniques, conducted on animals, exploring the relationship between VA and bacteria, fungi, parasites, or unspecified microorganisms, or focusing only on the vitamin’s capacity to stimulate the participants’ immune response without a confirmed viral infection.

### 2.2. Search Strategy

To reach adequate coverage of the clinical research conducted on the topic, two reviewers independently searched PubMed, Scopus, and Web of Science from database inception to 21 May 2021 using the following terms: virus OR disease OR infection OR viral AND retinoidal OR retinol OR vitamin a OR tretinoin OR retinoic acid. The string was adapted to fit the search criteria of each database (Supplementary Table S1). No filter was applied in the search strategy. Duplicate articles due to database overlap were removed, and the title and abstract of the collected records were screened. Studies that clearly did not meet the inclusion criteria were excluded. Full texts of potentially relevant articles were retrieved and independently examined by two researchers. Disagreements were resolved through discussion, and reasons for exclusion recorded. The reference lists of retrieved articles were also manually searched to identify other potentially relevant studies.

### 2.3. Data Collection and Synthesis

For each eligible study, two reviewers independently extracted the following information: first author, year of publication, country, virus family, characteristics of the target population, VA status at baseline, type and duration of the intervention, form and dosage of VA administered, follow-up time, area of evaluation (prevention or management of viral infections), main findings and side effects. As for the records investigating the VA effect on the management of viral infections, three categories were considered: virological response, immunological response, and clinical response. Articles providing data on different clinical outcomes but from people enrolled in the same trial were grouped. A narrative synthesis of the results was performed for each virus family. Two independent authors performed the quality assessment of the articles included in the systematic review using the revised Cochrane Risk-Of-Bias tool version 2 [19] for randomized studies. Discrepancies were resolved by consensus or by a third reviewer. Judgements on the quality of the studies followed the Cochrane guidelines [20].

## 3. Results

**Figure 1:**
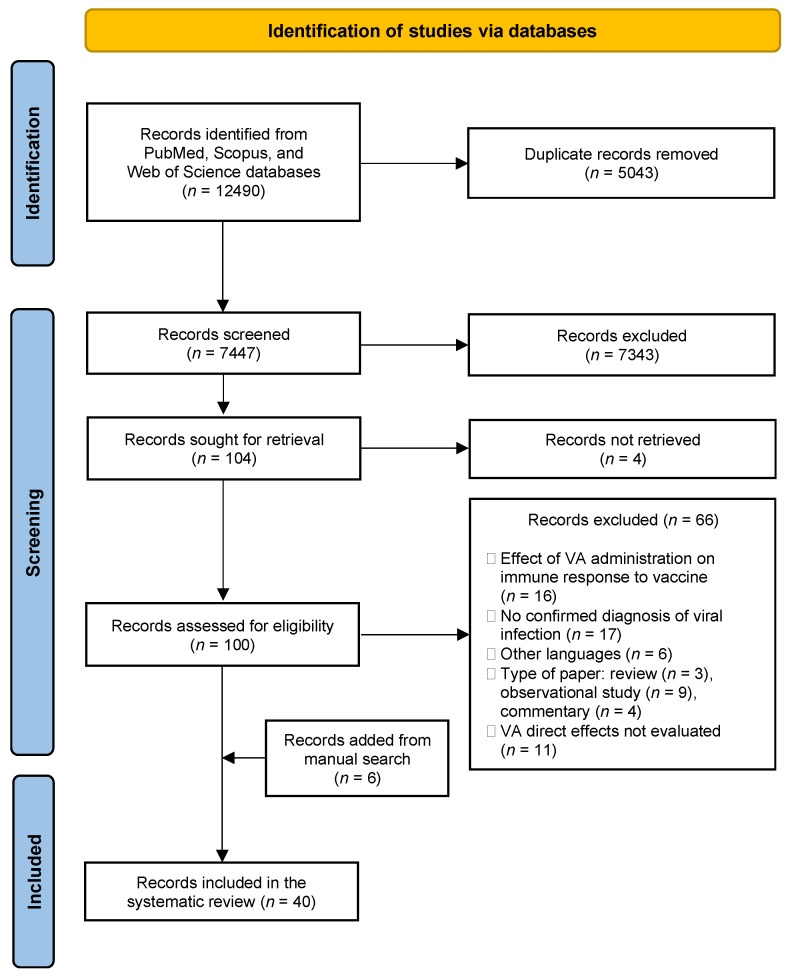
PRISMA flow diagram of the review process. VA: Vitamin A.

After the removal of duplicates, 7747 records resulted from the initial search (Figure 1). Screening by title and abstract selected 100 articles eligible for full-text analysis, from which 66 records were excluded with reasons. Six records were added to the previous 34 from the reference lists of relevant articles, for a total of 40 articles ultimately included in the systematic review.

### 3.1. Characteristics of the Included Studies by Virus Family

We found data on infections sustained by *Retroviridae* (human immunodeficiency virus 1, HIV-1; *n* = 17 articles), *Paramyxoviridae* (measles virus; *n* = 13 articles), *Pneumoviridae* (respiratory syncytial virus, RSV; *n* = 4 articles), *Papillomaviridae* (human papillomavirus, HPV; *n* = 3 articles), *Caliciviridae* (norovirus; *n* = 2 articles), and *Flaviviridae* (hepatitis C virus, HCV; *n* = 1 article) (Table 1). Studies investigating HIV-1 infection were published from 1995 to 2006 and analyzed data coming from ten RCTs; four RCTs were conducted in South Africa (*n* = 7 articles) [21,22,23,24,25,26,27], two RCTs in the United States (*n* = 2 articles) [28,29], two RCTs in Tanzania (*n* = 5 articles) [30,31,32,33,34], one RCT in Kenya (*n* = 2 articles) [35,36] and one RCT in Uganda (*n* = 1 article) [37], respectively. The target population mostly consisted of HIV-1 infected children (*n* = 4 RCTs) [21,25,31,37], followed by women aged 18–45 years (*n* = 2 RCTs) [29,35,36], pregnant women (*n* = 2 RCTs) [22,23,24,27,30,32,33,34], newly mothers (*n* = 1 RCT) [26], and drug users (*n* = 1 RCT) [28]. In one case [36], the target population was coinfected with HIV-1 and Herpes Simplex Virus 2. VA at baseline was evaluated in the majority of the studies: among the trials that reported the proportion of individuals enrolled with VA deficiency (*n* = 12), it ranged from 9.2% [26] to 100% [38,39], whereas in the trials that expressed the participants’ mean value of serum retinol concentration (*n* = 6 RCTs), it was always below the threshold for VA deficiency (i.e., <1.05 μmol/L or <30 μg/dL) [25,30,32,33,34,37,40,41], except from one case [42]. There was considerable heterogeneity in the intervention protocols. Oral VA administration ranged from 5000 IU every day for pregnant women [22,23,24,27,30,32,33,34] to one dose of 400,000 IU in newly mothers [26]. Follow-up time ranged from a few weeks [28,29] to 97 months [23]. As for the quality assessment, one article was judged at high risk of bias [25], ten articles had some concerns [21,22,23,24,26,27,29,31,35,36] and the remaining six studies had low risk of bias [28,30,32,33,34,37] (Supplementary Table S2).

Two articles [43,44] reported data from one trial that investigated the effects of VA administration on norovirus infections. It was conducted in Mexico and enrolled healthy children who were administered one dose of VA every two months for 15 months. The VA dosage depended on the child age. There were some concerns for its risk of bias (Supplementary Table S2).

One trial conducted in Japan [45] recruited patients with hepatitis C virus-related hepatocellular carcinoma, while three RCTs conducted in Greece [46], Mexico [47] and India [48], respectively, enrolled patients with genital or facial warts induced by HPV. They were orally administered peretinoin [45] or isotreninoin [46,47,48] each day for 3 [46,47,48] or 24 months [45], respectively. The risk of bias was low for the Japanese study [45], while the other trials were at high risk of bias [46,48] or had some concerns [47] (Supplementary Table S2).

As for *Pneumoviridae*, articles were published between 1988 and 1996 and the respective RCTs were conducted mostly in the United States (*n* = 2) [40,49], Australia (*n* = 1) [42] and Chile (*n* = 1) [41]. Three of them [40,41,49] enrolled children with RSV infection who received one dose of VA at hospital admission and were followed until hospital discharge, while one study recruited children with past RSV infection that were administered VA every week for 12 months [42]. Two RCTs were judged at high risk of bias [42,49], while the other two trials had some concerns [40,41] (Supplementary Table S2).

Lastly, six RCTs (*n* = 9 articles) [38,39,50,51,52,53,54,55,56], three cluster-randomized trials [57,58,59] and one community-based RCT [60] were published between 1987 and 2013 and investigated the effects of VA supplementation on measles infections among children. They were mostly conducted in India (*n* = 3 RCTs) [57,58,59] or South Africa (*n* = 2 RCTs) [8,51,52]. Large trials with over 4000 participants enrolled healthy children [54,55,57,58,59,60] who were supplemented with VA at different dosage once at birth [54,55] or at regular intervals (i.e., every week, every four months, or every six months) [57,58,59,60], while the other studies recruited children with severe measles who were given VA at baseline only [38,39,53] or multiple times during their hospital stay [8,50,51,52]. The follow-up time ranged from a few days for children admitted to hospital to 60 months [59]. Quality of articles was heterogeneous: six had low risk of bias [50,51,52,56,57,60], six had some concerns [38,39,53,54,55,59], and the last one was deemed at high risk of bias [58] (Supplementary Table S2).

**Table 1:** Characteristics of the studies included in the systematic review by virus family.

Virus Family	Author, Year	Country	Study Design	Population	VA Status at Baseline (Serum Retinol) *	VA Form and Dose	Frequency of VA Administration	Follow-Up Time	Risk of Bias
*Retroviridae*	Coutsoudis, 1995 [21]	South Africa	RCT	28 HIV-1 infected children born from HIV-1 infected women	NA	OA of retinyl palmitate: <3 months: 50,000 IU 3–12 months: 100,000 IU ≥12 months: 200,000 IU	One dose at month 1, 3, 6, 9 and 12	97 months	SC
	Coutsoudis, 1997 [22] (#)	South Africa	RCT	24 HIV-1 infected pregnant women	NA	OA of retinyl palmitate: 5000 IU and 200,000 IU	One dose every day and at delivery	1 week after delivery	SC
	Coutsoudis, 1999 [23] (#)			661 HIV-1 infected pregnant women	30.6% of women < 20 μg/dL			3 months after delivery	SC
	Kennedy, 2000 [27] (#) Kennedy-Oji, 2001 [24] (#)			312 HIV-1 infected pregnant women	66% of women < 30 μg/dL 37% of women < 20 μg/dL				SC SC
	Semba, 1998 [28]	United States	RCT	120 HIV-1 infected drug users	18.3% of patients < 1.05 μmol/L	OA of retinyl palmitate: 200,000 IU	One dose at baseline	4 weeks	Low
	Humphrey, 1999 [29]	United States	RCT	41 HIV-1 infected women aged 18–45 years	9.8% of women < 1.05 μmol/L	OA of retinyl palmitate: 300,000 IU	One dose at baseline	4 weeks	SC
	Baeten, 2002 [35] (§)	Kenya	RCT	400 HIV-1 infected women aged 18–45 years	58.5% of women < 30 μg/dL	OA of retinyl palmitate: 10,000 IU	One dose every day for 6 weeks	6 weeks	SC
	Baeten, 2004 [36] (§)			376 women aged 18–45 years coinfected with HIV-1 and HSV-2	58.2% of women < 30 μg/dL				SC
	Villamor, 2002 (a) [30] (δ)	Tanzania	RCT	1078 HIV-1 infected pregnant women	Mean value: 84–90 μmol/L	OA of retinyl palmitate: 5000 IU and 200,000 IU	One dose every day and at delivery	Until delivery	Low
	Fawzi, 2004 (a) [33] (δ)			1078 HIV-1 infected pregnant women	Mean value: 85–88 μmol/L			72 months	Low
	Fawzi, 2004 (b) [32] (δ)			852 HIV-1 infected pregnant women	Mean value: 25.1–26.5 μg/dL			Until delivery	Low
	Webb, 2009 [34] (δ)			626 HIV-1 infected pregnant women	Mean value: 82–89 μmol/L			12 months after delivery	Low
	Villamor, 2002 (b) [31]	Tanzania	RCT	47 HIV-1 infected children aged 6–60 months hospitalized for pneumonia	NA	OA of retinyl palmitate: <12 months: 100,000 IU ≥12 months: 200,000 IU	One dose on day 1, 2, at 4 and 8 months	12 months	SC
	Semba, 2005 [37]	Uganda	RCT	181 HIV-1 infected children aged 15 months	Mean value: 56–58 μmol/L	OA of 60 mg of retinol equivalent	One dose every 3 months for 9 months	21 months	Low
	Humphrey, 2006 [25]	South Africa	RCT	2,266 children born from HIV-1 infected women	Mean value: 94–1.01 μmol/L	OA of retinyl palmitate: 50,000 IU	One dose at delivery	24 months	High
	Zvandasara, 2006 [26]	South Africa	RCT	4,495 HIV-1 infected women post-partum	9.2% of women < 1.05 μmol/L	OA of retinyl palmitate: 400,000 IU	One dose at delivery	24 months	SC
*Caliciviridae*	Long, 2007 [43] Long, 2011 [44]	Mexico	RCT	127 healthy children aged 5–15 months	NA	OA of retinyl palmitate: <12 months: 20,000 IU ≥12 months: 45,000 IU	One dose every two months for 15 months	15 months	SC SC
*Flaviviridae*	Okita, 2014 [45]	Japan	RCT	377 patients with hepatitis C virus-related hepatocellular carcinoma	NA	OA of peretinoin: 600 mg/day or 300 mg/day	One dose every day for 24 months	48 months	Low
*Papillomaviridae*	Georgala, 2004 [46]	Greece	RCT	60 women aged 21–43 years with RCA of the cervix	NA	OA of isotretinoin: 0.5 mg/kg/day	One dose every day for 3 months	3 months	High
	Olguin-Garcıa, 2014 [47]	Mexico	RCT	31 patients with recalcitrant facial flat warts	NA	OA of isotreninoin: 30 mg/day	One dose every day for 3 months	3 months	SC
	Kaur, 2017 [48]	India	RCT	40 patients with multiple plane warts	NA	OA of isotretinoin: 0.5 mg/kg/day TA of isotretinoin: 0.05% per day	One dose every day for 3 months or until lesion clearance	4 months	High
*Pneumoviridae*	Pinnock, 1988 [42]	Australia	RCT	206 children aged 2–7 years with past RSV infection during infancy	Mean value: 37–40.8 μg/dL	OA of retinyl palmitate: 2000 IU	One dose every week for 12 months	12 months	High
	Breese, 1996 [40]	United States	RCT	239 children aged 1 month-6 years hospitalized with RSV infection	Mean value: 21.5–22.5 μg/dL	OA of retinyl palmitate: 1–5 months: 50,000 IU 6–11 months: 100,000 IU ≥12 months: 200,000 IU	One dose at hospital admission	Until hospital discharge	SC
	Dowell, 1996 [41]	Chile	RCT	180 children aged 1 month-6 years hospitalized with RSV infection	Mean value: 23–24 μg/dL	OA of retinyl palmitate: 1–5 months: 50,000 IU 6–11 months: 100,000 IU ≥12 months: 200,000 IU	One dose at hospital admission	Until hospital discharge	SC
	Quinlan, 1996 [49]	United States	RCT	32 children aged 2 months-5 years hospitalized with RSV infection	∼50% of children < 0.70 μmol/L	OA of retinyl palmitate: 100,000 IU	One dose at hospital admission	Until hospital discharge	High
*Paramyxoviridae*	Barclay, 1987 [50]	Tanzania	RCT	180 children with severe measles admitted to hospital	91% of children < 0.51 μmol/L	OA of retinyl palmitate: 200,000 IU	One dose at hospital admission and on day 2	Until hospital discharge	Low
	Hussey, 1990 [56]	South Africa	RCT	189 children with severe measles admitted to hospital	92% of children < 0.70 μmol/L	OA of retinyl palmitate: 400,000 IU	Half dose at hospital admission and on day 2	Until hospital discharge	Low
	Rahmathullah, 1990 [57]	India	CRT	15,419 children aged 6–60 months	37.5% of children < 0.70 μmol/L	OA of retinyl palmitate: 8333 IU	One dose every week for 12 months	12 months	Low
	Coutsoudis, 1991 [51] Coutsoudis, 1992 [52]	South Africa	RCT	60 children aged 4 months-2 years with severe measles admitted to hospital	90% of children < 0.70 μmol/L	OA of retinyl palmitate: <12 months: 100,000 IU ≥12 months: 200,000 IU	One dose at hospital admission, on day 2, day 8 and day 42	6 months	Low Low
	Ogaro, 1993 [53]	Kenya	RCT	294 children aged < 5 years with severe measles admitted to hospital	21% of children < 20 μg/dL	OA of retinyl palmitate: 1–5 months: 50,000 IU 6–11 months: 100,000 IU ≥12 months: 200,000 IU	One dose at hospital admission	Until hospital discharge	SC
	Agarwal, 1995 [58]	India	CRT	15,247 children aged < 6 years	NA	OA of retinyl palmitate: 1–6 months: 50,000 IU 7–72 months: 100,000 IU	One dose every 4 months for 12 months	24 months	High
	Rosales, 1996 [38] Rosales, 2002 [39]	Zambia	RCT	196 children aged 5–17 years with acute measles not requiring hospitalization	100% of children < 20 μg/dL	OA of retinol: 210 µmol retynil esters	One dose at baseline	1 month	SC SC
	Dollimore, 1997 [60]	Ghana	C-RCT	25,443 healthy children aged >6 months	NA	OA of retinyl palmitate: <12 months: 100,000 IU ≥12 months: 200,000 IU	One dose every 4 months for 24 months	24 months	Low
	Benn, 2008 [54] Diness, 2011 [55]	Guinea-Bissau	RCT	4345 healthy newborns	NA	OA of retinyl palmitate: 50,000 IU	One dose at birth	12 months	SC SC
	Awasthi, 2013 [59]	India	CRT	1,000,000 children aged < 6 years	NA	OA of retinyl acetate: 200,000 IU	One dose every 6 months for 60 months	60 months	SC

(#) or (δ) or (§): studies with the same symbol included participants from the same trial but with different number of participants and/or follow-up time. * Serum retinol concentration: <20 μg/dL, severe deficiency; <30 μg/dL, deficiency; <0.35 μmol/L, severe deficiency; <0.70 μmol/L, moderate deficiency; <1.05 μmol/L, deficiency. C-RCT: community-based Randomized Controlled Trial. CRT: Cluster Randomized Trial. IU: International Unit. NA: Not Assessed. OA: Oral Administration. RCA: recalcitrant condylomata acuminata. RCT: Randomized Controlled Trial. RSV: respiratory syncytial virus. SC: Some Concerns. TA: Topical Administration.

### 3.2. Main Findings by Virus Family

#### 3.2.1. *Retroviridae*

Most trials conducted on HIV-1-infected individuals compared VA administration to placebo only [21,22,23,24,25,27,28,29,31,35,36,37], while one RCT [30,32,33,34] compared VA to multivitamin without VA, multivitamin including VA, or placebo, respectively (Table 2). In one case [36], a subgroup analysis was performed among women with CD4 >200 cells/mm3. Consistent results were found for the virological response, for which it was not observed any difference in plasma or genital HIV-1 viral load [22,28,29,32,35] or in genital HSV DNA [36] between treated and untreated individuals. The immunological response showed similar results: VA administration did not seem to have any effect on CD4 cell count [24,27,28,29,35], CD8 cell count [29,35] or IL-1b levels [32]. Conversely, the clinical response was heterogeneous. The overall morbidity rates of gastrointestinal and HIV-related symptoms were not found to differ between VA supplemented and non-supplemented individuals [21,24,26,27,32,36,37], but a lower number of cause-specific clinic visits for a few conditions [26] or a lower incidence of diarrhea [21] were found in one study each, respectively. Among pregnant women, the occurrence of preterm births was lower in one case [23], but the maternal weight gain was similar between the two groups in two trials [24,27,30]. One trial [24,27] also reported a significant higher retention of post-partum weight gain in the group supplemented with VA, while another [34] mentioned a significant higher concentration of retinol, b-carotene, and a-carotene in breast milk. One study [31] reported a greater height gain among HIV-1 infected children supplemented with VA. Three out of four trials that quantified death rates did not report any VA beneficial effect in the long term [25,26,32]. Lastly, the only author that analyzed side effects did not find meaningful differences between treated and untreated individuals [29].

#### 3.2.2. *Caliciviridae*

The two articles [43,44] that analyzed the same trial on norovirus comparing VA to placebo reported different results in the prevention and clinical immunological response depending on the genogroup (Table 3). Precisely, whereas VA administration did not have any effect on the incidence of norovirus genogroup I (NoV-GI) infections, it seemed to prevent the occurrence of infections sustained by norovirus genogroup II (NoV-GII). Likewise, while the authors found a significantly higher fecal TNF-α and IL-4 concentration during NoV-GI infections among VA-supplemented patients, the MCP-1 and TNF-α fecal levels were lower in case of NoV-GII infections. By contrast, for both viruses a significantly higher duration of viral shedding was found together with a significant lower incidence of the associated diarrheal disease, but no difference in the occurrence of the associated fever was observed. Side effects were not evaluated.

#### 3.2.3. *Flaviviridae*

The only study on Flaviviridae, specifically on HCV, [45] compared two different VA dosages to placebo (Table 3); a greater recurrence free survival was found only in the group with the highest dosage. Mild or moderate side effects were reported in relation to VA administration.

#### 3.2.4. *Papillomaviridae*

The effects of VA on HPV infections were analyzed in three studies, two of which compared oral VA administration to placebo [46,47], while the third compared it to topical VA application [48] (Table 3). The outcome was clearance from facial or genital lesions, that was found significantly higher among patients treated with oral VA in all studies [46,47,48] similarly to the side effects, that were deemed mild or moderate in all articles [46,47,48].

**Table 2:** Main effects of Vitamin A (VA) administration in the management of individuals infected with human immunodeficiency virus type 1 (HIV-1).

Author, Year	Intervention	Management			Side Effects
		Virological Response	Immunological Response	Clinical Response/Others	
Coutsoudis, 1995 [21]	Group I: VA Group II: placebo	NA	NA	Significant lower diarrhea incidence in Group I Non-significant difference in diarrhea duration, respiratory infections, rash, and mean weight gain Non-significant difference in overall morbidity	NA
Coutsoudis, 1997 [22] (#)	Group I: VA Group II: placebo	Non-significant difference in HIV-1 plasma viral load	NA	NA	NA
Coutsoudis, 1999 [23] (#)		NA	NA	Significant lower incidence of preterm births in Group I Non-significant difference in mean birth weight	NA
Kennedy, 2000 [27] (#) Kennedy-Oji, 2001 [24] (#)		NA	Non-significant difference in CD4 cell count	Non-significant difference in hemoglobin concentration Non-significant difference in the frequency of HIV-related symptoms Non-significant difference in maternal weight gain Significant higher retention of post-partum weight gain in Group I	NA
Semba, 1998 [28]	Group I: VA Group II: placebo	Non-significant difference in HIV-1 plasma viral load	Non-significant difference in CD4 cell count	NA	NA
Humphrey, 1999 [29]	Group I: VA Group II: placebo	Non-significant difference in HIV-1 plasma viral load (as mean, median and change) at each time point	Non-significant difference in median percentage of CD4 cells and of CD8 cells that are CD38+ at each time point	NA	Non-significant difference
Baeten, 2002 [35] (§)	Group I: VA Group II: placebo	Non-significant difference in median vaginal and plasma HIV-1 viral load	Non-significant difference in CD4 and CD8 cell count	NA	NA
Baten, 2004 [36] (§)	Group I: VA Group II: placebo Only women with CD4 >200 cells/mm^3^: Subgroup I: VA Subgroup II: placebo	Non-significant difference in the detection of genital HSV DNA or mean HSV DNA load between Group I and Group II and between Subgroup I and Subgroup II	NA	Non-significant difference in genital ulceration between Group I and II	NA
Villamor, 2002 (a) [30] (δ)	Group I: VA + BC Group II: MVI without VA Group III: MVI with VA+ BC Group IV: placebo	NA	NA	Non-significant difference in maternal weight gain outcomes overall or during the third trimester Significant lower risk of low total weight gain in Group I + III vs. Group II	NA
Fawzi, 2004 (a) [33] (δ)		NA	NA	Non-significant difference in progression to stage 4 or death from AIDS-related causes between Group I and IV Non-significant difference in risk of thrush, oral ulcers, painful tongue or mouth, and fatigue between Group I and IV Non-significant difference in risk of other oral or gastrointestinal manifestations between Group I and IV	NA
Fawzi, 2004 (b) [32] (δ)		Non-significant difference in HIV-1 plasma or genital viral load	Non-significant difference in IL-1b level	NA	NA
Webb, 2009 [34] (δ)		NA	NA	Significant higher concentration of breast milk retinol, b-carotene, and a-carotene in Group I vs. IV	NA
Villamor, 2002 (b) [31]	Group I: VA Group II: placebo	NA	NA	Significant greater height gain in Group I	NA
Semba, 2005 [37]	Group I: VA Group II: placebo	NA	NA	Significant lower mortality in Group I Non-significant difference in the prevalence of diarrhea, cough fever, ear discharge, blood in stool, need for hospitalization	NA
Humphrey, 2006 [25]	Group I: VA Group II: placebo	NA	NA	Significant higher infection-or-death rates in Group I at 12 months Non-significant difference in mortality rate at 24 months	NA
Zvandasara, 2006 [26]	Group I: VA Group II: placebo	NA	NA	Non-significant difference in overall and cause-specific mortality Non-significant difference in the overall number of sick clinic visits Significant lower number of cause-specific clinic visits for malaria, vaginal infection, pelvic inflammatory diseases, and cracked or bleeding nipples Non-significant difference in need for hospitalization	NA

(#) or (δ) or (§): studies with the same symbol have included participants from the same trial. BC: beta-carotene. HSV: Herpes Simplex Virus. MVI: multivitamins. NA: not assessed.

**Table 3:** Main effects of Vitamin A (VA) oral administration in the prevention and management of viral infections by virus family.

Author, Year	Intervention	Prevention	Management			Side Effects
			Virological Response	Immunological Response	Clinical Response/Others	
*Caliciviridae*						
Long, 2007 [43] Long, 2011 [44]	Group I: VA Group II: placebo	Non-significant difference in incidence of NoV-GI infections Significant lower incidence in NoV-GII infections in Group I	Significant higher duration of NoV-GI and NoV-GII shedding in Group I	Significant higher fecal TNF-α and IL-4 concentration in Group I during NoV-GI infections Significant lower fecal MCP-1 and TNF-α concentration in Group I during NoV-GII infections	Significant lower incidence of all NoV-associated diarrheal disease and diarrhea associated with GI and GII infections in Group I Non-significant difference in the incidence of NoV-associated fever	NA
*Flaviviridae*						
Okita, 2014 [45]	Group I: VA (600 mg) Group II: VA (300 mg) Group III: placebo	NA	NA	NA	Significant higher RFS in Group I vs. III Non-significant difference in RFS in Group II vs. III	Mild, moderate, or serious side effects in relation to VA dosage
*Papillomaviridae*						
Georgala, 2004 [46]	Group I: VA Group II: placebo	NA	NA	NA	Significant higher clearance of cervical lesions in Group I	Mild or moderate side effects in Group I
Olguin-Garcıa, 2014 [47]	Group I: VA Group II: placebo	NA	NA	NA	Significant higher clearance of facial lesions in Group I	Mild or moderate side effects in Group I
Kaur, 2017 [48]	Group I: VA Group II: topical VA 0.05% in gel	NA	NA	NA	Significant greater clearance of lesions (number and timing) in Group I	Mild or moderate side effects in both groups
*Pneumoviridae*						
Pinnock, 1988 [42]	Group I: VA Group II: placebo	Non-significant difference in number of episodes and duration of respiratory illness	NA	NA	NA	NA
Breese, 1996 [40]	Group I: VA Group II: placebo	NA	NA	NA	Non-significant difference in oxygen requirement, need for steroids, ribavirin, ICU care or mechanical ventilation Significant longer hospital stay and lower proportion of patients discharged within 48 h in Group I	Non-significant difference in side effects occurrence
Dowell, 1996 [41]	Group I: VA Group II: placebo	NA	NA	NA	Non-significant difference in duration of hospitalization, oxygen requirement and time to resolve hypoxemia Significant more rapid resolution of tachypnea and shorter duration of hospitalization in Group I among children with severe hypoxemia at admission	None
Quinlan, 1996 [49]	Group I: VA Group II: placebo	NA	NA	NA	Non-significant difference in daily severity score, hospital stay, need for ICU care or oxygen requirement	None

ICU: intensive care unit. IL-4: interleukin 4. MCP-1: monocyte chemoattractant protein-1. NA: Not Assessed. NoV-GI: norovirus genogroups I. NoV-GII: norovirus genogroups II. RFS: recurrence free survival. TNF-α: Tumor necrosis factor alfa.

#### 3.2.5. *Pneumoviridae*

Four authors analyzed RSV infections comparing VA to placebo [40,41,42,49] (Table 3). No significant effect was found between the two groups in the only article that studied the prevention of episodes of respiratory illness [42], while the results were mixed in relation to the clinical management. Specifically, while all three studies [40,41,49] reported no significant difference in need for supplemental treatments, one study [40] found a longer hospital stay among patients treated with VA, one study [49] reported a similar length of stay between the two groups and the last study [41] described a shorter duration of hospital stay but among children with severe hypoxemia at admission only. Lastly, out of the three articles that investigated side effects, two of them did not report any adverse reaction [41,49], while Breese and colleagues did not find any significant difference between treated and untreated children [40].

#### 3.2.6. *Paramyxoviridae*

All the studies performed on *Paramyxoviridae* were focused on measles virus. Specifically, six trials focused on measles compared VA to placebo [38,39,50,51,52,54,55,56,60], one of which differentiated children marginally VA-deficient from those VA-sufficient [38,39]; three trials compared a combination of VA and Vitamin E to Vitamin E only [53,57,58], and one trial had four arms: VA, albendazole, VA plus albendazole and placebo [59] (Table 4). Large trials on the prevention of measles occurrence or measles-specific mortality did not indicate any benefit from VA supplementation [54,55,57,58,59,60]. The immunological response did not show any meaningful finding apart from higher IgG antibodies in treated children in one study [51,52]. Among children with severe measles, three RCTs investigated the mortality rate [50,53,56], but only two reported a protective effect among supplemented children [50,56]. Some positive results were found for a few measles-related complications, such as pneumonia occurrence or duration [51,52,56], especially among VA-deficient children [38,39], severe diarrhea [53,56], or otitis media [53], whereas other aspects did not seem to differ [51,52,53,56]. Only one trial evaluated side effects, with no adverse reaction mentioned [56].

**Table 4:** Main effects of Vitamin A (VA) oral administration in the prevention and management of infections sustained by measles virus.

Author, Year	Intervention	Prevention	Management		Side Effects
			Immunological Response	Clinical Response/Others	
Barclay, 1987 [50]	Group I: VA Group II: placebo	NA	NA	Significant lower mortality in Group I	NA
Hussey, 1990 [56]	Group I: VA Group II: placebo	NA	NA	Significant lower mortality in Group I Significant lower duration of pneumonia and diarrhea in Group I Significant lower measles croup occurrence in Group I Non-significant difference in airway intervention, herpes stomatitis occurrence, and need for intensive care	None
Rahmathullah, 1990 [57]	Group I: VA + VE Group II: VE	Non-significant difference in measles-specific mortality	NA	NA	NA
Coutsoudis, 1991 [51] Coutsoudis, 1992 [52]	Group I: placebo Group II: VA	NA	Significant higher measles IgG antibodies in Group II at day 8 and 42 Non-significant difference in IL-2 and complement values at day 2, day 8 and day 42	Significant lower duration of pneumonia or recovery time in Group II at day 8 Significant lower IMS in Group II at day 8 Non-significant difference in duration of diarrhea or fever at day 8 Significant lower IMS in Group II at day 42 Significant higher weight gain in Group II at day 42 Significant lower IMS in Group II at 6 months Non-significant difference in weight gain at 6 months	NA
Ogaro, 1993 [53]	Group I: VA + VE Group II: VE	NA	NA	Non-significant difference in overall occurrence of diarrhea, laryngotracheobronchitis, or pneumonia Significant lower occurrence of severe diarrhea in Group I Significant lower occurrence of otitis media in Group I Significant lower duration of diarrhea in Group I for those who had already it on admission Non-significant difference in mortality	NA
Agarwal, 1995 [58]	Group I: VA + VE Group II: VE	Non-significant difference in measles-specific mortality	NA	NA	NA
Rosales, 1996 [38] Rosales, 2002 [39]	Marginally VA-deficient children: Group I: VA Group II: placebo VA-sufficient children: Group III: VA Group IV: placebo	NA	Non-significant difference in serum CRP concentration	Significant lower risk of developing pneumonia in Group I + III Significant lower risk of relapsing in Group I + III Significant lower pneumonia occurrence in Group I vs. II Non-significant difference in pneumonia occurrence between Group III and IV	NA
Dollimore, 1997 [60]	Group I: VA Group II: placebo	Non-significant difference in measles occurrence Non-significant difference in measles-specific mortality	NA	NA	NA
Benn, 2008 [54] Diness, 2011 [55]	Group I: VA Group II: placebo	Non-significant difference in measles occurrence Non-significant difference in need for hospitalization or mortality for measles-related complications	NA	NA	NA
Awasthi, 2013 [59]	Group I: VA Group II: albendazole Group III: VA + albendazole Group IV: placebo	Non-significant difference in measles-specific mortality	NA	NA	NA

VE: Vitamin E. NA: Not Assessed. IL: Interleukin. IMS: Integrated Morbidity Score. CRP: C-Reactive Protein.

## 4. Discussion

The spread of the COVID-19 pandemic has renewed the debate on the use of natural agents in preventing and managing viral infections [17]. Recent studies have shown benefits after the administration of a few vitamins [14,61], but no conclusive evidence on VA is available to date. Indeed, other studies have already synthesized the effects of VA in relation to specific outcomes, such as mortality, blindness, mother-to-child HIV transmission [62,63,64,65], but to the best of our knowledge a collection of evidence on its direct effects in relation to viral infections was still lacking.

In our review, a high proportion of studies investigated the management of infectious diseases, in line with our inclusion criterion that required a confirmation of the viral infection, more easily obtained in chronic conditions. Almost half of the studies focused on HIV-1, and most of them enrolled pregnant women or children living in African countries. This was not unexpected, given that nowadays the sub-Saharan region accounts for nearly 61% of new HIV cases [66]. However, we did not find any effect in relation to VA and virological, immunological, or clinical response, even though some weak but positive results were mentioned concerning a few HIV-related complications. This lack of efficacy may contribute to explain why studies that investigated HIV were conducted in a well-defined period and stopped after 2006. Not to mention the introduction of the first triple combination of antiretroviral drugs in a single tablet, a fundamental step toward an effective and generally well-tolerated option for the management of HIV infection [67], that may have caused an interest loss in searching for supplemental treatments for this disease.

The potential preventive role of VA was evaluated in the child population only. Despite in vitro studies demonstrating an effect in modulating the immune response that could reduce host susceptibility to infections [68,69], no convincing evidence in reducing the occurrence of infections sustained by norovirus, RSV, or measles virus was found. In this regard, it is not a coincidence that most trials were conducted in developing countries where, despite the progress made by the global vaccination campaigns performed by the World Health Organization [70], measles vaccination coverages are still largely insufficient [71]. However, we found encouraging effects in the management of a few measles-related aspects. For these reasons, given the global resurgence in measles cases observed since 2016 due to vaccine hesitancy phenomenon [72,73] and the significant disruptions to immunization services in many parts of the world during the COVID-19 pandemic [74], VA supplementation could be considered a beneficial intervention to reduce some complications.

As for the other viral infections, no clear conclusion could be drawn in relation to VA and the management of patients infected by HCV, norovirus, or RSV. Interestingly, while the RCTs focusing on the first two viruses were conducted in the past 15 years, suggesting that some effects of VA are still an object of research interest, most trials focused on RSV were concentrated in 1996 and stopped thereafter, probably the results of a large virus outbreak that occurred in the United States [75] that grabbed the scientific attention in those years. However, results were contradictory even within the same study, meaning that more research is needed to better understand the potential role of VA in providing care to these individuals, especially considering that some countries have started to document reemergent RSV epidemics after its disappearance in 2020 because of the precautions taken during the COVID-19 pandemic [76]. Lastly, consistent results were described in the reduction of some HPV-related lesions, even though the low study quality poses some challenges in the interpretation of these findings. Hence, since the global coverage of HPV vaccination is suffering with an estimated rate at 15% in 2019 [77], implying that a large proportion of individuals are still susceptible to genital or facial warts, VA supplementation may represent an interesting area for further investigations.

This study has some strengths and limitations. The main strength is the systematic collection of evidence on the topic. Indeed, to the best of our knowledge, this is the first systematic review that investigated the direct effects of VA administration in the prevention and management of confirmed viral infections. The limitations to the current review are mostly related to the primary studies included. Since most of them were conducted in low- and middle-income countries and/or enrolled great proportions of individuals with VA deficiency, the generalizability of our findings may be limited. In addition, given that only a few studies have been published recently, updated evidence is lacking, especially for some virus families. Furthermore, a large heterogeneity in the recruitment and treatment protocols was found, limiting the comparability of the results and the opportunity to provide a quantitative synthesis even within the same viral family. Lastly, the quality of the trials was variable, making the interpretation of the results more difficult. For these reasons, further studies are needed to better investigate the potential benefits of VA oral administration in relation to viral infections, using a common pre-established daily dosage of VA, a standardized time of administration and a fixed follow-up period. Moreover, since a confirmed viral infection was an inclusion criterion, it is possible that we may not have included a few data on the effects of VA on the infections in which the etiological agent was not specified. However, it was impossible to be sure about the infectious source given the low specificity of the symptoms, and our focus was limited to the vitamin’s antiviral activity.

## 5. Conclusions

Despite its relatively safe profile, our systematic review did not find meaningful results between VA oral supplementation and the prevention of viral infections. By contrast, encouraging results were described for the management of some viral diseases, according to which VA supplemented individuals had a better prognosis and improved outcomes, such as for HPV lesions or some measles-related complications. Given that they are both vaccine-preventable diseases and considering the decline in immunization coverages registered during the COVID-19 pandemic, VA could play an interesting role in the management of these infections, especially in low-middle income countries where the vaccination campaigns may be difficult to implement. However, further research is needed to better investigate the potential benefits of VA oral administration in relation to viral infections, possibly using standardized recruitment and treatment protocols.

## Data Availability

Not applicable.

## Supplementary Materials

The following supporting information can be downloaded at: https://www.mdpi.com/article/10.3390/nu14194081/s1, Table S1: Search strategies used in the systematic review; Table S2: Revised Cochrane risk-of-bias tool for randomized trials (RoB2).
