# Supplementary material for: What Are the Effects of Vitamin A Oral Supplementation in the Prevention and Management of Viral Infections? A Systematic Review of Randomized Clinical Trials
Source: Nutrients. 2022 Oct 1;14(19):4081. doi: 10.3390/nu14194081 (PMC9572963; doi:10.3390/nu14194081)
Supplement: Supplementary file 1 [file nutrients-14-04081-s001.zip › nutrients-1937908-supplementary.pdf]

Table S1. Search strategies used in the systematic review.

PubMed	(("virus diseases"[MeSH Terms] OR ("virus"[All Fields] AND "diseases"[All Fields]) OR "virus diseases"[All Fields] OR ("viral"[All Fields] AND "infection"[All Fields]) OR "viral infection"[All Fields]) OR virus OR viruses) AND ("retinoids"[MeSH Terms] OR "retinoidal"[All Fields] OR "retinoids"[All Fields] OR "retinoid"[All Fields] OR ("vitamin a"[MeSH Terms] OR "vitamin a"[All Fields]) OR ("tretinoin"[MeSH Terms] OR "tretinoin"[All Fields] OR ("retinoic"[All Fields] AND "acid"[All Fields]) OR "retinoic acid"[All Fields]))
Scopus	TITLE-ABS-KEY (("virus disease" OR (virus AND disease) OR (viral AND infection) OR "viral infection") AND (retinoidal OR retinoid OR "vitamin a" OR tretinoin OR (retinoic AND acid) OR "retinoic acid"))
Web Of Science	((("virus disease" OR (virus AND disease) OR (viral AND infection) OR "viral infection" OR virus) AND (retinoidal OR retinoid OR "vitamin a" OR tretinoin OR (retinoic AND acid) OR "retinoic acid")))

Table S2. Revised Cochrane risk-of-bias tool for randomized trials (RoB2).

Author, year	DOMAIN 1	DOMAIN 2		DOMAIN 3	DOMAIN 4	DOMAIN 5	
	Risk of bias arising from the randomization process	Risk of bias due to deviations from the intended interventions (effect of assignment to intervention)	Risk of bias due to deviations from the intended interventions (effect of adhering to intervention)	Missing outcome data	Risk of bias in measurement of the outcome	Risk of bias in selection of the reported result	Overall risk of bias
Coutsoudis, 1995	Low	Some Concerns	Low	Low	Some Concerns	Some Concerns	Some Concerns
Coutsoudis, 1997	Some Concenrs	Some Concerns	Some Concerns	Some Concerns	Low	Low	Some Concerns
Coutsoudis, 1999	Some Concerns	High	Some Concerns	Some Concerns	Low	Low	Some Concerns
Kennedy, 2000	Some Concerns	Some Concerns	Low	Low	Some Concerns	Low	Some Concerns
Kennedy-Oji, 2001	Some Concerns	Low	Some Concerns	Low	Some Concerns	Low	Some Concerns
Semba, 1998	Some Concerns	Some Concerns	Low	Low	Low	Low	Low
Humphrey, 1999	Low	High	High	Some Concerns	Some Concerns	Some Concerns	Some Concerns
Baeten, 2002	Some Concerns	Some Concerns	Some Concerns	Low	Low	Some Concerns	Some Concerns
Baeten, 2004	Some Concerns	Some Concerns	Some Concerns	Some Concerns	Low	Low	Some Concerns
Villamor, 2002 (a)	Low	Low	Some Concerns	Some Concerns	Low	Low	Low
Fawzi, 2004 A	Some Concerns	Some Concerns	Some Concerns	Low	Some Concerns	Some Concerns	Low
Fawzi, 2004 B	Low	Low	Some Concerns	Low	Some Concerns	Low	Low
Webb, 2009	Low	Low	Some Concerns	Low	Some Concerns	Low	Low
Villamor, 2022 (b)	Low	Some Concerns	Some Concerns	Some Concerns	Some Concerns	Low	Some Concerns
Semba, 2005	Low	Low	Some Concerns	Low	Low	Some Concerns	Low
Humphrey, 2006	Low	High	High	High	Some Concerns	Some Concerns	High
Zvandasara, 2006	Some Concerns	Some Concerns	Low	Some Concerns	Low	Low	Some Concerns
Long, 2007	Some Concerns	Some Concerns	Low	Some Concerns	Some Concerns	Low	Some Concerns
Long, 2011	Some Concerns	Some Concerns	Low	Low	Low	Low	Some Concerns
Okita, 2014	Low	Low	Low	Low	Low	Low	Low
Georgala, 2004	Some Concerns	Some Concerns	Some Concerns	High	High	High	High
Olguin-Garcıa, 2014	Low	Low	Some Concerns	Some Concerns	Some Concerns	Some Concerns	Some Concerns
Kaur, 2017	Some Concerns	High	High	Some Concerns	Some Concerns	Low	High
Pinnock, 1988	Some Concerns	Low	Low	Some Concerns	Some Concerns	High	High
Breese, 1996	Low	Low	Some Concerns	Some Concerns	Some Concerns	Some Concerns	Some Concerns
Dowell, 1996	Low	Low	Some Concerns	Some Concerns	Some Concerns	Low	Some Concerns
Quinlan, 1996	High	High	Some Concerns	Some Concerns	High	Some Concerns	High
Barclay, 1987	Low risk	Low risk	Low risk	Low risk	Low risk	Low risk	Low risk
Hussey, 1990	Low risk	Low risk	Low risk	Low risk	Low risk	Low risk	Low risk
Rahmathullah, 1990	Some concerns	Low risk	Low risk	Low risk	Low risk	Low risk	Low risk

Coutsoudis, 1991	Low risk	Low risk	Low risk	Low risk	Low risk	Low risk	Low risk
Coutsoudis, 1992	Low risk	Low risk	Low risk	Low risk	Low risk	Low risk	Low risk
Ogaro, 1993	Some concerns	Low risk	Low risk	Low risk	Some concerns	Low risk	Some concerns
Agarwal, 1995	Some concerns	Some concerns	Some concerns	Some concerns	Some concerns	Some concerns	High risk
Rosales, 1996	Low risk	Low risk	Low risk	Some concerns	Some concerns	Some concerns	Some concerns
Rosales, 2002	Low risk	Low risk	Low risk	Some concerns	Some concerns	Some concerns	Some concerns
Dollimore, 1997	Low risk	Some concerns	Some concerns	Low risk	Low risk	Low risk	Low risk
Benn, 2008	Some concerns	Low risk	Some concerns	Some concerns	Some Concerns	Some Concerns	Some Concerns
Diness, 2011	Some concerns	Low risk	Low risk	Low risk	Low risk	Some concerns	Some concerns
Agawashi, 2013	Low risk	Some concerns	Some concerns	Low risk	Low risk	Low risk	Some concerns
